# Fluctuations of Epstein-Barr Virus Serological Antibodies and Risk for Nasopharyngeal Carcinoma: A Prospective Screening Study with a 20-Year Follow-Up

**DOI:** 10.1371/journal.pone.0019100

**Published:** 2011-04-22

**Authors:** Su-Mei Cao, Zhiwei Liu, Wei-Hua Jia, Qi-Hong Huang, Qing Liu, Xiang Guo, Teng-Bo Huang, Weimin Ye, Ming-Huang Hong

**Affiliations:** 1 State Key Laboratory of Oncology in Southern China, Department of Epidemiology, Clinical Trial Study Center, Cancer Center, Sun Yat-sen University, Guangzhou, China; 2 State Key Laboratory of Oncology in Southern China, Department of Experimental Research, Cancer Center, Sun Yat-sen University, Guangzhou, China; 3 Sihui Cancer Institute, Sihui, China; 4 State Key Laboratory of Oncology in Southern China, Department of Nasopharyngeal Carcinoma, Cancer Center, Sun Yat-sen University, Guangzhou, China; 5 Department of Medical Epidemiology and Biostatistics, Karolinska Institutet, Stockholm, Sweden; City of Hope National Medical Center and Beckman Research Institute, United States of America

## Abstract

**Background:**

The impact of variation of Epstein-Barr virus (EBV) antibody titers before the development of nasopharyngeal carcinoma (NPC) is still unclear. We analyzed the fluctuations of antibodies against EBV before histopathological diagnosis to assess the risk of NPC and aimed to provide a reliable basis for screening in high risk populations.

**Methods:**

This study was based on a population-based screening program in Sihui County in Guangdong Province of China. A total of 18,986 subjects were recruited in 1987 and 1992, respectively. Baseline and repeated serological tests were performed for IgA antibodies against EBV capsid antigen (VCA/IgA) and early antigen (EA/IgA). Follow-up until the end of 2007 was accomplished through linkage with population and health registers. Cox proportional hazards regression model was used to estimate the relative risk of NPC in association with EBV antibodies. Time-dependent receiver operating characteristic curve (ROC) analysis was used to further evaluate the predictive ability.

**Results:**

A total of 125 NPCs occurred during an average of 16.9 years of follow-up. Using baseline information alone or together with repeated measurements, serological levels of VCA/IgA and EA/IgA were significantly associated with increased risks for NPC, with a striking dose-response relationship and most prominent during the first 5 years of follow-up. Considering the fluctuant types of serological titers observed during the first three tests, relative risk was highest among participants with ascending titers of EBV VCA/IgA antibodies with an adjusted hazard ratio (HR) of 21.3 (95% confidence interval [CI] 7.1 to 64.1), and lowest for those with decreasing titers (HR = 1.5, 95% CI 0.2 to 11.4), during the first 5 years of follow-up. Time-dependent ROC analysis showed that VCA/IgA had better predictive performance for NPC incidence than EA/IgA.

**Conclusion:**

Our study documents that elevated EBV antibodies, particularly with ascending titers, are strongly associated with an increased risk for NPC.

## Introduction

Nasopharyngeal carcinoma (NPC) is a rare malignancy in most populations of the world, with incidence rates lower than 1 per 100,000 person-years [1]. However, among populations in the southern parts of China and Southeast Asia, where NPC is more endemic than any parts of the world, the incidence rates are as high as 20 to 50 per 100,000 person-years, especially in Cantonese-speaking men residing in Guangdong Province and Hong Kong of Southern China [1]–[5]. Salted-fish consumption [6]–[10], Epstein-Barr virus (EBV) infection [11]–[19] and genetic susceptibility [20]–[23] are considered to be the major risk factors that contribute to such a distinguished geographic distribution for this cancer.

Although it has not been addressed thoroughly, several pieces of evidence suggest that EBV infection is strongly associated with the occurrence of NPC, especially the undifferentiated subtype of non-keratinising carcinoma [14], the most common histopathological type in southern China according to WHO classification [24]. As early as in 1966, elevation of antibodies against EBV antigens in NPC patients was observed [25]. In 1973, presence of EBV genomes was demonstrated in epithelial NPC cells [18] and EBV-related antigens were detected in the tumor cells of all NPC patients [26]. Subsequently, the expression of the viral genome in non-keratinising NPC has been studied extensively in areas with NPC epidemic [14], [16], [27] and major types of viral expression proteins have been found, e.g., EBNA1 [28]–[30], LMP1 [15], [31]–[34] and LMP2A [31]. Moreover, in case-control studies, NPC cases showed significantly higher antibody titers to EBV antigens than controls in several retrospective studies [11], [19], [35]–[38]. However, in these studies, serological results were mainly based on blood specimens collected after the diagnosis of NPC, thus these findings might not clarify the critical issue of EBV replication in relation to occurrence of NPC. To avoid the concern of potential reversal causality, blood samples should be collected long time before clinical evidence appears.

To our knowledge, to date three prospective population-based studies, which were based in Guangxi province, Zhongshan City and Taiwan [39]–[45], have been conducted to explore the relation between EBV antibodies and NPC onset. Findings from these studies suggest that IgA antibodies against EBV capsid antigen (VCA/IgA) is a biomarker associated with the risk of NPC development and using this marker as a screening tool for NPC is feasible [43]–[44], [46]–[47]. Moreover, IgA antibody against EBV early antigen (EA/IgA) is a highly specific marker, which is usually assayed simultaneously with VCA/IgA for the diagnosis of NPC [48]–[50]. Although the previous studies have several advantages, the dose-response relationship between EBV antibody titers and NPC risk is not yet clear. In fact, antibody levels are always changing during the progression of NPC, however, the association between fluctuant pattern of EBV antibodies and NPC risk has not been explored. Further, the predictive abilities of these markers in population cohort have not been reported. Moreover, it is possible that seronegative subjects go through seroconversion before NPC develops, but data regarding this issue are still lacking.

To further evaluate the relationship between EBV infection and NPC risk, we used repeated serological data from a large prospective population-based screening study in Sihui County, which is located along the Pearl River in Guangdong Province, China, one of the high-risk areas of NPC. In 1987, a screening study using antibodies against EBV as biomarkers was initiated, aiming at improving early detection rate and reducing the mortality rate of the cancer. Here we analyzed the results after a 20-year follow-up from this cohort to clarify the role of EBV infection in relation to risk of NPC in the high incidence area of Southern China, especially the dose-response relationship and the serological antibody fluctuation over time long time before NPC occurrence.

## Results

After 301,933 person-years of follow-up, 125 cases of incident NPC were identified one year after recruitment into the study cohort (Table 1), which rendered an age-adjusted incidence rate of 68.0 per 100,000 person-years for males and 25.0 per 100,000 person-years for females. The overall baseline sero-prevalence rates of VCA/IgA and EA/IgA antibodies were 7.16% (1,318/18,411) and 0.24% (45/18,411), respectively. The age-adjusted incidence rate of NPC per 100,000 person-years was 29.4 among subjects seronegative for VCA/IgA, 188.2 among those seropositive for VCA/IgA but seronegative for EA/IgA, and 617.4 among those seropositive for both markers.

**Table 1:** Baseline characteristics, crude and age-adjusted incidence rates (1/100,000 person-years) of nasopharyngeal carcinoma (NPC) among 18,411 participants in the Sihui NPC screening cohort, Sihui, Guangdong, China (1987–2007).

	Total subjects	NPC patients	Person-years	Crude-incidence rate (1/100,000 person-years)	Age-adjusted incidence rate (1/100,000 person-years)*
	(n = 18,411)	(n = 125)	(n = 301,933)		
Gender					
Male	7,078	79	115,712	68.3	68
Female	11,333	46	186,221	24.7	25
Age at entry					
30–39 yr	8,653	49	144,634	33.9	–
40–49 yr	5,309	43	85,963	50.0	–
50–59 yr	4,449	33	71,336	46.3	–
Area of residence (town)					
Longjiang	3,412	27	64,734	41.7	42.7
Jingkou	4,363	40	82,213	48.7	48.6
Jianggu	5,162	31	75,457	41.1	38.8
Didou	5,474	27	79,529	33.9	33.2
Status of serological tests at baseline†					
VCA/IgA (−)	17,093	83	280,798	29.6	29.4
VCA/IgA (+)					
EA/IgA (−)	1,262	38	20,300	187.2	188.2
EA/IgA (+)	45	4	671	596.3	617.4
Missing	11	0	164	0	0

*Standardized to the distribution of person-time experienced by the entire cohort (5-year age groups).

† After subjects were tested positive for IgA antibodies against EBV capsid antigen (VCA/IgA), tests for IgA antibodies against EBV early antigen (EA/IgA) were also performed. Eleven subjects did not have enough sera for EA/IgA test.

**Figure 1:**
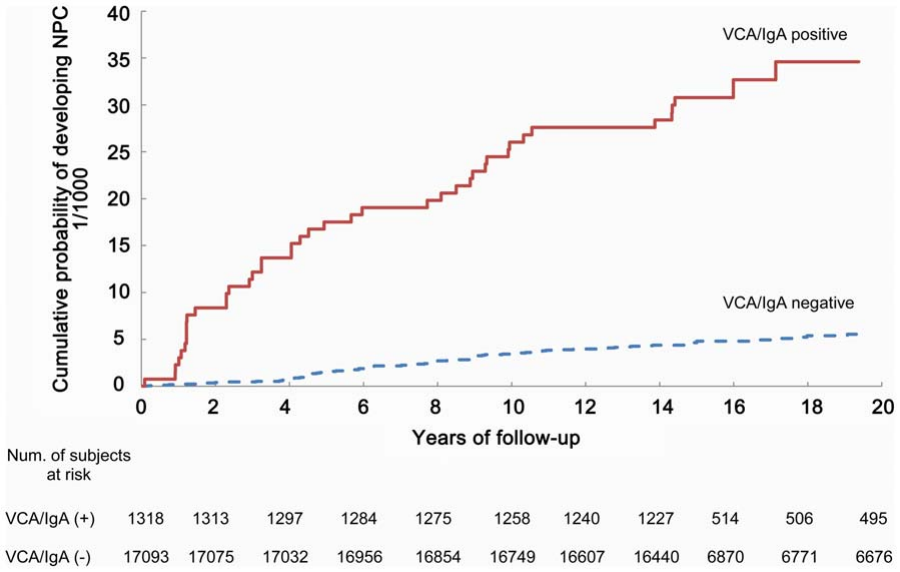
Cumulative probability of developing nasopharyngeal carcinoma (NPC) during follow-up among 18,411 subjects, according to baseline levels of IgA antibodies against EBV capsid antigen (VCA/IgA) (1987–2007).

We confirmed that level of VCA/IgA antibodies was a strong risk indicator for NPC (Table 2). For attained follow-up duration up to 5 years, compared with seronegative subjects, the adjusted HRs were 6.7 (95% CI 2.7 to 16.3), 9.4 (95% CI 4.0 to 21.9), 22.5 (95% CI 8.6 to 59.1) and 41.9 (95% CI 16.0 to 110.2) for subjects whose baseline VCA/IgA antibody titers were 1∶5, 1∶10, 1∶20 and ≥1∶40, respectively. For attained follow-up duration more than 5 years, the HRs decreased but still showed a clear dose-response relationship, with the corresponding HRs of 0.9 (95% CI 0.2 to 3.6), 6.4 (95% CI 3.2 to 13.2), 6.3 (95% CI 2.0 to 20.4) and 18.0 (95% CI 7.2 to 45.2), respectively. After 18 years of follow-up, the cumulative probability of developing NPC was 34.6‰ for subjects seropositive for VCA/IgA and 5.4‰ for those seronegative for VCA/IgA, respectively (Figure 1). Among subjects seropositive for VCA/IgA, compared with VCA/IgA seronegative stratum, those seropositive also for EA/IgA had significantly higher HRs compared with those seronegative for EA/IgA, and the estimates during the first 5 years of follow-up were consistently higher than those after 5 years of follow-up (Table 2).

**Table 2:** Hazard ratios (HRs) and 95% confidence intervals (CIs) for nasopharyngeal carcinoma associated with baseline or repeated levels of antibodies against Epstein-Barr virus antigens.

Serological status at baseline		Entire cohort*				Sub-cohort§
		Num. of subjects	Num. of cases	Adjusted HRs (95% CIs) by attained follow-up duration†		Adjusted HR (95% CIs)
				≤5 years	>5 years	
VCA/IgA (−)		17,093	83	1.0 (reference)	1.0 (reference)	1.0 (reference)
VCA/IgA (+)						
1∶5		585	8	6.7 (2.7, 16.3)	0.9 (0.2, 3.6)	3.7 (0.9, 16.0)
1∶10		501	16	9.4 (4.0, 21.9)	6.4 (3.2, 13.2)	3.0 (0.7, 12.7)
1∶20		144	8	22.5 (8.6, 59.1)	6.3 (2.0, 20.4)	11.2 (4.2, 29.8)
≥1∶40		88	10	41.9 (16.0, 110.2)	18.0 (7.2, 45.2)	32.6 (17.5, 60.8)
Combination of two markers						
VCA/IgA	EA/IgA‡					
1∶5∼1∶10	(−)	1041	21	7.0 (3.4, 14.1)	2.8 (1.4, 5.5)	2.6 (0.8, 8.7)
1∶5∼1∶10	(+)	37	3	48.1 (11.1, 209.0)	9.9 (1.4, 72.1)	19.3 (2.4, 157.7)
≥1∶20	(−)	221	17	27.8 (12.9, 59.9)	11.1 (5.3, 23.5)	16.1 (8.4, 30.9)
≥1∶20	(+)	8	1	88.7 (11.7, 673.3)	N/A∥	95.6 (44.4, 206.1)

*In this analyis, all 18,411 subjects were included, and only baseline serological information was used. HRs were adjusted for sex, age at entry (30–39, 40–49, 50–59 years) and residency by town.

† Since the proportional hazards assumption did not hold, results were presented separately by attained follow-up duration (≤5 vs >5 years).

‡ EA/IgA infomation from 11 subjects were missing, and these subjects were excluded from this analysis.

§ This analysis included only 7890 subjects, who had at least two serological tests for VCA/IgA antibodies. Among them, 54 NPCs were identifid during follow-up. HRs and 95%CIs were derived from time-dependent Cox proportional hazards regression model, adjusted for sex, age at entry (30–39, 40–49, 50–59 years) and residency by town.

∥ No NPC was found in this stratum.

Using repeated serological results of 7,890 subjects in the course of NPC screening and treating them as time-dependent variables in the Cox regression model, the results were similar to those based on baseline test only (Table 2). Subjects with highest VCA/IgA titers (≥1∶40) had the highest adjusted HR (32.6, 95% CI 17.5 to 60.8) when compared with VCA/IgA baseline negative group. For the group with titers of ≥1∶20 for VCA/IgA and positive for EA/IgA, compared with the group baseline seronegative for both markers, the adjusted HR was as high as 95.6 (95% CI 44.4 to 206.1). In order to quantitatively measure fluctuation of antibody titers and its effect on cancer outcome, we also treated baseline measurement as constant variable, and the differences between each repeated test and baseline measurement as time-varying variables, adjusted for age, sex, and town. Results similarly indicated that subjects with increasing serological titers for VCA/IgA had a higher risk of developing NPC. Those subjects with one unit of increase (equal to double the titers at baseline) or more during follow-up rendered an adjusted HR of 12.4 (95% CI 5.4 to 28.5) compared with the category with stable VCA/IgA titers, while the corresponding HR was 1.8 (95% CI 0.6 to 5.4) for less than 1 unit of increase during follow-up.

**Figure 2:**
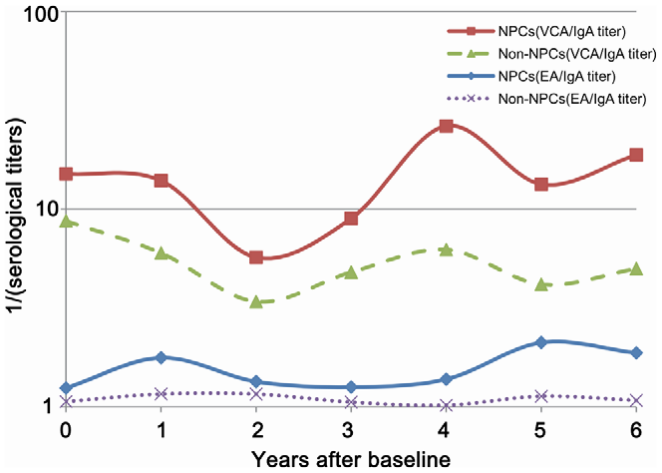
Variation of IgA antibodies against Epstein-Barr virus capsid (VCA/IgA) or early antigen (EA/IgA) over time in 42 subjects who developed nasopharyngeal carcinoma (NPC) during follow-up and in 1,276 subjects who were NPC-free by the end of follow-up, among the group baseline seropositive for VCA/IgA (1,318 subjects). Note: Geometric means of serolgocial titres in each follow-up year were calculated. Specifically, the numbers of tests were 42, 37, 18, 19, 5, 16 and 11 for baseline, 1st year, 2nd year, 3rd year, 4th year, 5th year and 6th or more year of follow-up, respectively in NPC group. The numbers of tests were 1276, 1217, 615, 813, 287, 1008 and 708 for baseline, 1st year, 2nd year, 3rd year, 4th year, 5th year and 6th or more year of follow-up, respectively in NPC-free group.

Figure 2 shows the fluctuation of EBV VCA/IgA and EA/IgA antibody titers over time by outcome among subjects seropositive for VCA/IgA at baseline. Although both serological biomarkers fluctuated over time, NPC group had consistently higher antibody levels than those of NPC-free subjects. Among 962 participants seropositive for VCA/IgA at baseline and with 3 or more tests, 129 participants were classified into Ascending group, and among them 6 cases of NPC were detected, yielding an adjusted HR of 21.3 (95% CI 7.1 to 64.1) in the first 5 years of follow-up and 7.8 (95% CI 1.9 to 33.2) during the later follow-up duration, compared with baseline seronegative participants. Among 426 subjects who belonged to Stable group, 11 cases of NPC were identified, yielding an adjusted HR of 6.2 (95% CI 2.2 to 17.8) in the first 5 years of follow-up and 5.5 (95% CI 2.5 to 12.2) in later period of follow-up (Table 3). In contrast, the relative risks for Descending group were much lower than the other two groups. In the first 5 years of follow-up, the adjusted HR was only 1.5 and statistically non-significant (Table 3).

**Table 3:** Adjusted hazard ratios (HRs) and 95% confidence intervals (CIs) for nasopharyngeal carcinoma (NPC) by fluctuant type of serological status during follow-up among 962 subjects seropositive for VCA/IgA antibodies against EBV capsid antigen compared to 17,093 seronegative subjects at baseline*.

Serological status at baseline	No. of subjects	No. of cases	Adjusted HRs (95% CIs)†	
			Attained follow-up duration‡	
			≤5 years	>5 years
VCA/IgA (−)	17,093	83	1.0 (reference)	1.0 (reference)
VCA/IgA (+)				
Ascending	129	6	21.3 (7.1, 64.1)	7.8 (1.9, 33.2)
Stable	426	11	6.2 (2.2, 17.8)	5.5 (2.5, 12.2)
Descending	407	6	1.5 (0.2, 11.4)	2.9 (1.2, 7.4)

*Among the totally 1318 subjects seropositive for VCA/IgA antibodies at baseline, 356 had fewer than 2 repeated tests. Thus these subjects could not be classified into any fluctuant group, thus were excluded from current analysis. Among them, 19 NPC cases were indentified.

† Adjusted for sex, age at entry (30–39, 40–49, 50–59 years) and residency by town.

‡ Since the proportional hazards assumption did not hold, results were presented separately by attained follow-up duration (≤5 vs >5 years).

Furthermore, 6,554 subjects seronegative in 1987 (or 1992) baseline VCA/IgA test and free of NPC during follow-up were retested for VCA/IgA antibodies in 1997. Among 438 subjects who were seropositive for VCA/IgA in 1997, 6 subjects developed NPC in the follow-up period from year 1998 to 2007. Whereas, only 10 cases of NPC were found among 6,116 subjects who were seronegative both in 1987 (or 1992) and 1997. The corresponding HR for NPC in association with seroconversion to VCA/IgA positivity was 9.0 (95% CI 3.2 to 24.8).

**Figure 3:**
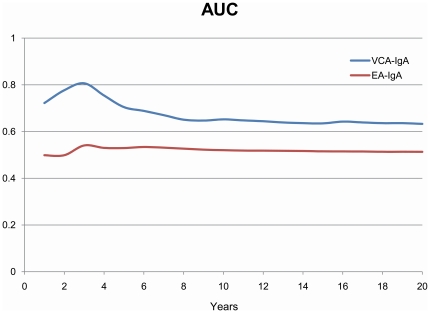
Time-dependent ROC curve analysis for nasopharyngeal carcinoma (NPC) prediction according to baseline levels of IgA antibodies against EBV capsid antigen (VCA/IgA) and early antigen (EA/IgA) (ordinal categorical variable). ROC, receiver operating characteristic; AUC, area under the ROC curve, NPC, nasopharyngeal carcinoma.

AUCs during follow-up from time-dependent ROC curve analysis were shown in Figure 3. VCA/IgA showed good predictive performance over the follow-up duration, and was consistently better than EA/IgA. The sensitivity at 3^rd^ year of follow-up for VCA/IgA was 66.7%, 50.1% and 33.4%, respectively, when the cutoff value was set to 1∶5, 1∶10, or 1∶20; the corresponding specificity was 92.9%, 96.1% and 98.8%, respectively. Compared with VCA/IgA, the sensitivity for EA/IgA was lower, but the specificity was higher for different cutoff values (Table S1). The AUC at 3^rd^ year was 0.807 for VCA/IgA and 0.541 for EA/IgA. The predictive ability of VCA/IgA in females was consistently better than in males and did not vary importantly by different age group (data not shown).

## Discussion

This large population-based prospective serological screening study conducted in the high risk area of NPC in Guangdong province has confirmed the strong association between serological EBV antibody markers and NPC risk. Particularly in the first 5 years of follow-up, a clear dose-response relationship between EBV antibody titers and NPC risk was demonstrated. These results indicated that the anti-EBV sero-markers are mainly short-term risk predictors in the NPC development, although caution is needed when interpreting results by follow-up duration because of lead time bias, as baseline seropositive group was followed up much closer than seronegative group. However, even after 5 years of follow-up this association still existed, although the strength of association decreased with follow-up duration. Our results are consistent with previous reports. In several mass screening studies conducted in Wuzhou and Cangwu, China [42]–[45], Zeng et al. reported that the NPC annual incidence rate for VCA/IgA(+) persons was 2-fold to 81-fold the rate in age-group-matched general population. In another study conducted in Taiwan [39]–[40], [51], elevated VCA/IgA and neutralizing antibodies against EBV DNAse were found to be highly specific sero-markers for predicting NPC, with a 20-fold increase in NPC risk for subjects seropositive for VCA/IgA antibodies, and a 30-fold increase for those seropositive for both biomarkers. Recently, a study conducted in Zhongshan City [41] reported that subjects seropositive for VCA/IgA antibodies had a relative risk of 5.8 for developing NPC, as compared with the general screening population.

Although there is so far no evidence from randomized clinical trial to support the screening of NPC using the EBV serological markers [52], in fact, it is very popular to conduct NPC screening since the 1970's in the southern China [43]–[44], [53], and the two markers of VCA/IgA and EA/IgA are most commonly used. Correct interpretation of repeated test results and prediction of NPC risk is important for providing clinical counseling, which is critical for early detection and prognosis of the malignancy [54]–[55]. To our knowledge, our study is the first to illustrate the relationship between fluctuation of EBV seromarkers and NPC outcomes. Those subjects with ascending VCA/IgA antibody titers tended to have higher risks and shorter time to develop NPC, compared to those with a descending pattern. Therefore, for these patients, closer follow-up and rigorous clinical examination are recommended.

The nature of the association between EBV and NPC is still under debate. Persistent high titers of EBV-specific antibodies could be the results of aberrant viral gene expression [56]. In our study, seroconversion into VCA/IgA positive before NPC development was observed. Further, in Zhongshan study [41], a serologic window before NPC diagnosis when antibodies levels are raised and sustained was also reported, with a mean duration of 37 months. These results suggest that positive seroconversion might be a key step in the pathogenesis of NPC and the elevation of EBV antibody titers may play an important role in the process of NPC development.

Advantages of our study include the relatively large sample size and a long-term follow-up. In addition, 96.8% (121/125) of NPC cases were histopathologically confirmed. The unique information of repeated tests enabled us to study the fluctuation of anti-EBV sero-markers before the onset of NPC, which have not been reported by previous studies. However, the current study still has some limitations. One main concern is the lacking of information of other environmental risk factors, which precluded the possibilities for controlling the potential confounding effects from these risk factors. In addition, this study was conducted in a high-risk area, and our results might not be applicable to other areas with medium or low risk for NPC. Last, although this study is up to date the largest, the small number of NPC cases made estimates in some strata imprecise.

In conclusion, titers of antibodies against EBV may represent the level of EBV replication. VCA/IgA and EA/IgA antibodies are useful seromarkers for NPC screening in high risk areas. In future NPC screening programs, close monitoring of subjects seropositive for VCA/IgA antibodies are highly recommended.

## Materials and Methods

### Study Population

**Figure 4:**
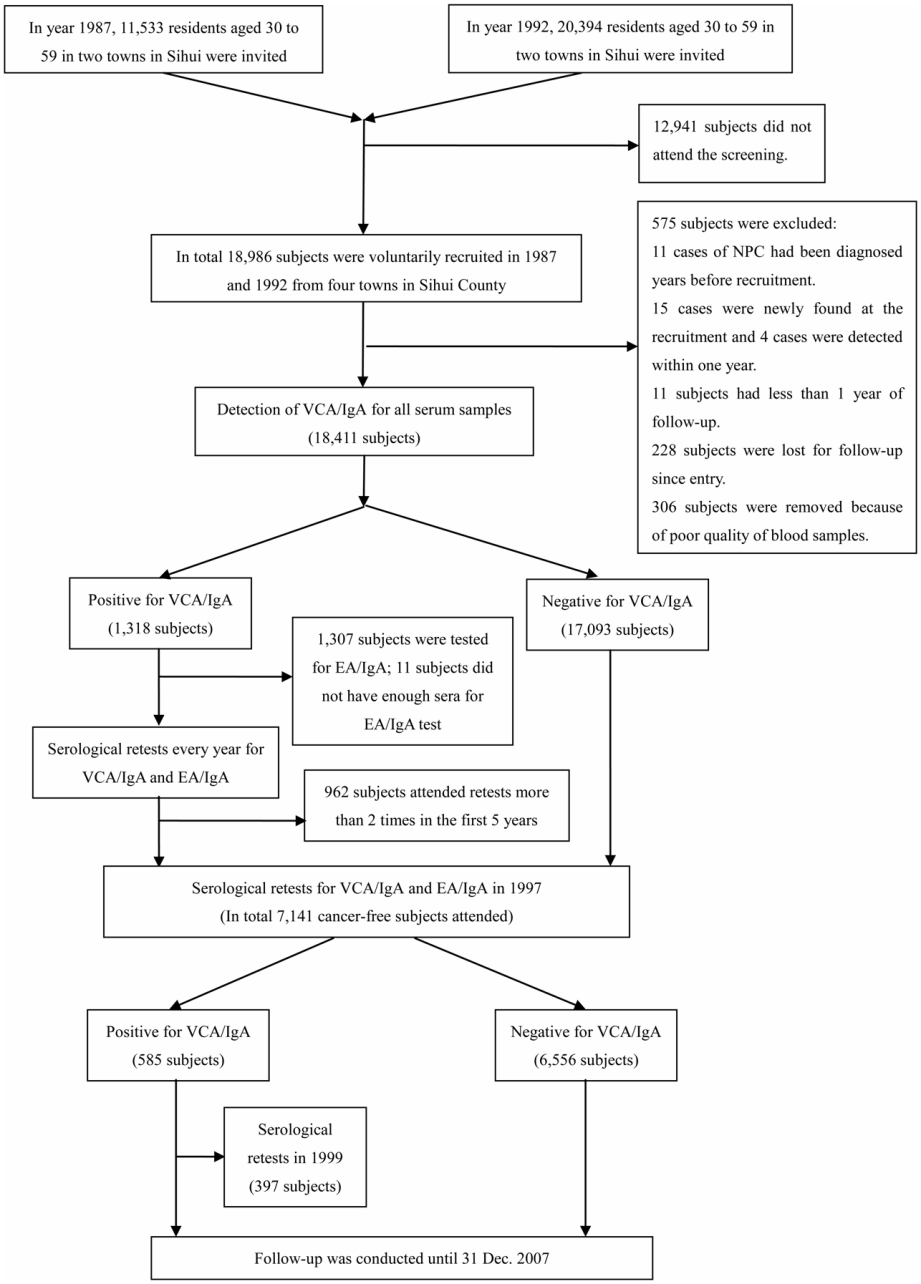
Protocol of Serological Tests for Anti-Epstein-Barr Virus Antibodies in the Nasopharyngeal Carcinoma Screening Cohort from 1987 to 2007 in Sihui, Guangdong, China.

In 1987, the Chinese National Programs for Science and Technology Development during the Seventh Five-Year Plan Period of China was firstly launched in two towns from Sihui County located in Guangdong province in the southern China, which aimed at improving early detection of NPC through serological screening tests for VCA/IgA and EA/IgA. During the period of National Eighth Five-Year Plan, we extended our research areas to four towns (Figure 4). Staff in Sihui Cancer Institute and local village committee advertised and invited the residents to attend this screening program. The inclusion criteria were those who were Cantonese, residing in Sihui County, without recorded history of NPC and with good physical condition and mental health. The exclusion criteria were those who had severe cardiovascular, liver or kidney diseases, with a recorded NPC or not residing in Sihui County. Finally, 18,986 subjects, ages 30–59, were voluntarily recruited in 1987 and 1992, respectively from four towns of the Sihui County.

Medical staff from Sun Yat-sen University Cancer Center (SYSUCC), Sihui Cancer Institute and local hospitals formed research teams and visited the four towns, including Didou, Jianggu, Longjiang and Jingkou, to collect subjects' general socio-demographic information (birthday, gender, occupation, marital status, etc.) and blood samples. Written protocol documents had been sent to each local screening towns and villages. The local health of bureau and village committees have approved this screening (including using oral informed consents). All regional hospitals, village clinics and residents were informed about the details of the study and participating in this screening program was voluntary. Normally, the residents did not have any physical examinations before participating this screening program. Since Chinese Government did not require written informed consent before 1999, and a lot of local residents could not read written documents, oral informed consents were obtained instead.

Serological test for VCA/IgA was first performed. If the result was positive, the serological test for EA/IgA was further introduced. We also offered medical services such as otorhinolaryngologic and neck lymphatic examinations to the participants. If we found abnormal conditions, such as tubercle, coarse voice, ulcer in the nasopharynx, enlarged lymph nodes in the upper part of neck or serological tests of obvious abnormalities, fiberoptic endoscopy and pathological biopsy were performed by otorhinolaryngologists to diagnose NPC.

Eleven cases of NPC had been diagnosed several years before screening, 15 cases were newly found, and 4 cases were detected within one year after recruitment. Moreover, 11 subjects had less than 1 year of follow-up, and 228 subjects were lost to follow-up since entry. The other 306 subjects were removed due to poor quality of blood samples. Therefore, 575 subjects were excluded, leaving a total of 18,411 subjects for the final cohort. In year 1997, we re-contacted all subjects in this cohort to conduct serological tests. However, 8 subjects moved out of Sihui County. 143 died of diseases, accidents or unknown causes, 51 cases of NPC were identified during follow-up, and 11,068 subjects declined to participate. Finally, 7,141 subjects free of NPC were included in this re-test.

### Ethics Statement

Our study group had strictly abided by the principles of Helsinki Declaration and International Ethical Guidelines for Biomedical Research Involving Human Subjects developed by the Council for International Organizations of Medical Sciences (CIOMS) in collaboration with the World Health Organization (WHO). The baseline screening program was approved by Sun Yat-sen Medical University Cancer Center (later the Sun Yat-sen University Cancer Center). We promised to fully inform the subjects of possible adverse reactions, risk, discomfort and inconvenience during the screening study, and as well as treatment program with a possible compensation package. We made sure that all of the participants in this screening program were voluntary, and the subjects' medical documents (including the demographic data, testing results and diseases) to be saved securely. Permission to use the data from the screening program as well as further follow-up has been granted by the Institutional Ethics Review Board of Sun Yat-sen Medical University Cancer Center (No. YP2009169). Any published reports involved in the study wouldn't reveal the subjects' identification.

### Serologic Analysis

Each participant was asked to donate a 3 ml blood sample, which was collected into one Vacutainer tube without heparin. Blood samples were allowed to stay at room temperature for a maximum of 6 hours before being processed in a laboratory. Serum samples were then separated, divided into two tubes and stored at −20°C at the local hospital. One week after collection, sera were transported to Sun Yat-sen University Cancer Center (SYSUCC). All tests were performed in the laboratory of SYSUCC. EBV-specific VCA/IgA antibodies were measured using an immunoenzymic assay described previously [42]. Those with VCA/IgA titer ≥1∶5 were defined as seropositive group, and their levels of antibodies were further classified into four sub-groups (1∶5, 1∶10, 1∶20, ≥1∶40) according to the maximum dilution of serum. For the seropostive group, the EBV specific EA/IgA antibody levels were further measured using an immunoenzymic assay by Raji cell line [57]. Then the subjects were informed of the serological test results and advised for regular follow-up tests to examine the fluctuation of VCA/IgA and EA/IgA antibodies. The follow-up tests were performed every year for a total of up to 10 times during 1988 and 1999 (Figure 4). In SYSUCC, a regular quality control procedure for the assays has been used since 1980s [58]–[59]. Briefly, in order to ensure the reliability of the serological results, we used a pooled serological sample as our control sample. The coefficient of variation (CV) of the assay over 8 years (1993–2000) for VCA/IgA and EA/IgA was 8.37% and 8.47%, respectively.

### Definition of Antibody Fluctuation Pattern

**Figure 5:**
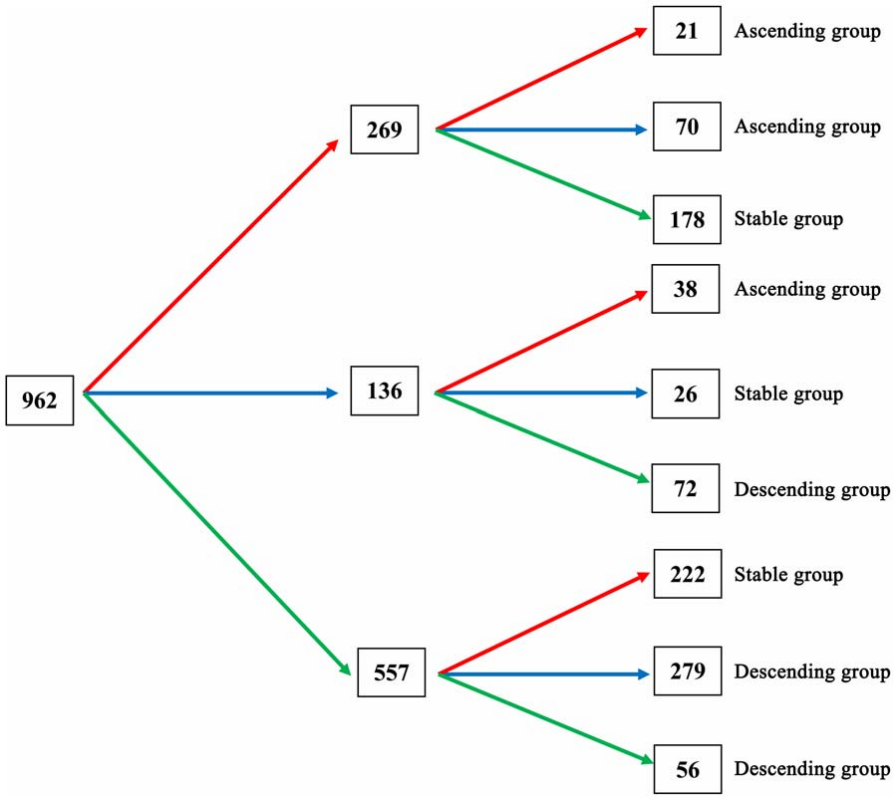
Graph of displaying different fluctuation patterns in 962 subjects seropositive for baseline VCA-IgA test and with at least 2 repeated tests during the first 5 years of follow-up. The red arrow shows that titers of VCA/IgA were higher than those in the previous test. The blue arrow shows that titers of VCA/IgA were equal to those in the previoust test. The green arrow shows that titers of VCA/IgA were lower than those in the previous test. Therefore, according to the definition, 129 (21+70+38) subjects were classified into the Ascending group, 407 (72+279+56) into the Descending group and 426 (178+26+222) into the Stable group. VCA/IgA, IgA antibodies against EBV capsid antigen; EA/IgA, IgA antibodies against EBV early antigen. **Definition: Ascending group:** Subjects whose repeated antibody titres were equal or higher than those in the previous test and the titres in the 3rd test were higher than the baseline. **Descending group:** Subjects whose repeated antibody titres were equal or lower than those in the previous test and the titres in the 3rd test were lower than the baseline. **Stable group:** The rest of subjects.

In total, among subjects who were seropositive at recruitment (1,318), 962 (73.0%) had undergone at least 2 repeated follow-up tests during the first 5 years of follow-up. We further classified them into three subgroups, i.e. Ascending, Stable or Descending, by using the first three VCA/IgA results (Figure 5). Subjects whose repeated antibody titers were equal or higher than those in the previous test and the titers in the 3rd test were higher than the baseline were classified into Ascending group (129 subjects). Subjects whose repeated antibody titers were equal or lower than those in the previous test and the titers in the 3rd test were lower than the baseline were classified into Descending group (407 subjects). The rest were classified as Stable group (426 subjects).

### Follow-up and Identification of Nasopharyngeal Carcinoma Cases

In Sihui County, a cancer registry was established in 1978, based on a three-level cancer prevention network, which was described previously [3]. Cancer cases were reported by local general practitioners, who provide health care for residents in villages, to the regional hospital of each town (approximately once each month). And then, health specialists assigned by Sihui Cancer Research Institute collected the reports and recorded information into pre-designed cards. For each incident cancer case, information collected includes registration identification number (ID), medical ID, Chinese Identity Card Number (unique for each resident), ICD code (9th or 10th version), name, sex, birth date, occupation, ethnicity, residence address, phone number, cancer site, base of diagnosis, and pathological report if available (date of diagnosis, names of hospital and physician in-charge). To ensure the completeness of cancer report, death certificates provided by the local public security bureau and hospitals were also reviewed. The percentage of ‘death certificate only’ cases was 5% during 1987–2007. Up to 90.3% of NPC cases were diagnosed by pathohistological review [3].

Furthermore, for correct censoring, dates of death were obtained from the Causes of Death Register and the Rosters of Village Committee, and dates of emigration came from the local public security bureau and the Rosters of Village Committee.

### Statistical Analysis

Person-years of follow-up for each cohort member were counted from the date of one year after recruitment to the date of NPC diagnosis, date of death or emigration, date of loss for follow-up, or December 31, 2007, whichever occurred first. Age-adjusted incidence rates by gender, area of residence and baseline EBV serological antibodies were standardized to distribution of person-time experienced by the entire cohort (5-year age groups). Kaplan-Meier method was used to derive the cumulative probability of NPC occurrence by baseline serostatus for VCA/IgA antibodies.

Hazard ratios (HRs) and their corresponding 95% confidence intervals (95% CIs) derived from Cox proportional hazards regression models were employed to explore the association between baseline EBV VCA/IgA and EA/IgA antibodies and the risk of NPC. The assumption of proportional hazards was tested using graphical method as well as the method based on Schoenfeld's residuals [60]. It was indicated that this assumption was not met for follow-up duration, as well as for age at entry and area of residence. Therefore, results were presented separately by attained follow-up duration (≤5 vs >5 years), and adjusted for sex (as covariate in the regression model), age at entry, and area of residence (as stratification variables).

In total, 7,890 subjects had undergone at least two serological tests for EBV VCA/IgA antibodies. Therefore, to account for time-varying exposure status, time-dependent Cox proportional hazards regression models were used to explore NPC risk in association with EBV infection. The first model used repeated serological test results as time-varying variables. To quantitatively measure the effect of antibody fluctuation on NPC risk, the second model included baseline measurement as constant variable, and the differences between each repeated test and baseline measurement as time-varying variables. The difference was calculated as, log_2_(maximum dilution of the repeated test) – log_2_(maximum diluation of the baseline test).

Geometric means of antibody titers at baseline and at each year of follow-up were calculated for baseline seropositive subjects to display the variation of antibody titers over time. Moreover, to explore the relation between fluctuant pattern of EBV-specific VCA/IgA antibodies and the risk of NPC, we classified 962 subjects seropositive for baseline VCA-IgA tests and with at least 2 repeated tests during the first 5 years of follow-up, into 3 subgroups according to different fluctuant patterns, i.e. “Ascending”, “Stable” or “Descending” (Figure 5). Cox proportional hazards regression model was used to analyze the relation between fluctuant type and NPC risk, using 17,093 subjects seronegative for VCA/IgA at baseline as reference group. Cox proportional hazards regression model was also used to calculate the relative risk of NPC in association with seroconversion for VCA/IgA positivity among 6,554 subjects who were seronegative for VCA/IgA at baseline and had been retested for EBV antibodies in year 1997.

To evaluate the predictive performance of VCA/IgA and EA/IgA, we employed the time-dependent receiver operating characteristics (ROC) curve for censored data and the area under the ROC curve (AUC) as the criterion [61]–[62]. Larger AUC indicates better predictability of time to event. AUC of 0.5 indicates no predictive ability, whereas a value of 1 represents perfect predictive ability. The “survivalROC” package for time-dependent ROC curve estimation in the open-source statistical software R (http://www.R-project.org) was used for the analysis.

All the statistical analyses, unless otherwise noted, were performed using SAS version 9.2 (SAS Institute, Cary, NC, USA). All statistical tests were two-sided, and a p value<0.05 was considered as statistical significant.

## Supporting Information

Table S1 Sensitivity, specificity and AUC in year 3, year 5 and year 10 of follow-up for screening of nasopharyngeal carcinoma using anti-EBV biomarkers.
(DOC)
