# Supplementary material for: Fluctuations of Epstein-Barr Virus Serological Antibodies and Risk for Nasopharyngeal Carcinoma: A Prospective Screening Study with a 20-Year Follow-Up
Source: PLoS One. 2011 Apr 22;6(4):e19100. doi: 10.1371/journal.pone.0019100 (PMC3081347; doi:10.1371/journal.pone.0019100)
Supplement: Table S1 — Sensitivity, specificity and AUC in year 3, year 5 and year 10 of follow-up for screening of nasopharyngeal carcinoma using anti-EBV biomarkers. (DOC) [file pone.0019100.s001.doc]

**Table S1. Sensitivity, specificity and AUC in year 3, year 5 and year 10 of follow-up for screening of nasopharyngeal carcinoma using anti-EBV biomarkers.**

| Serological status at baseline | Sensitivity (%) | | |  | Specificity (%) | | |  | AUC | | |
| --- | --- | --- | --- | --- | --- | --- | --- | --- | --- | --- | --- |
|  | Year 3 | Year 5 | Year 10 |  | Year 3 | Year 5 | Year 10 |  | Year 3 | Year 5 | Year 10 |
| Cutoff for VCA/IgA |  |  |  |  |  |  |  |  |  |  |  |
| 1:5 | 66.7 | 47.0 | 36.6 |  | 92.9 | 92.9 | 93.0 |  | 0.807 | 0.705 | 0.652 |
| 1:10 | 50.1 | 34.7 | 28.1 |  | 96.1 | 96.1 | 96.1 |  | 0.807 | 0.705 | 0.652 |
| 1:20 | 33.4 | 20.4 | 15.1 |  | 98.8 | 98.8 | 98.8 |  | 0.807 | 0.705 | 0.652 |
| Cutoff for EA/IgA |  |  |  |  |  |  |  |  |  |  |  |
| 1:5 | 8.3 | 6.1 | 4.3 |  | 99.8 | 100.0 | 100.0 |  | 0.541 | 0.529 | 0.520 |
| 1:10 | 8.3 | 6.1 | 3.2 |  | 99.9 | 100.0 | 100.0 |  | 0.541 | 0.529 | 0.520 |
| 1:20 | 4.2 | 2.0 | 1.1 |  | 99.9 | 100.0 | 100.0 |  | 0.541 | 0.529 | 0.520 |
| Combination of two markers |  |  |  |  |  |  |  |  |  |  |  |
| VCA/IgA(+)* & EA/IgA(-) | 66.7 | 47.0 | 36.6 |  | 93.0 | 93.0 | 93.0 |  | 0.801 | 0.701 | 0.649 |
| VCA/IgA(+)* & EA/IgA(+) | 8.3 | 6.1 | 4.3 |  | 99.8 | 99.8 | 99.8 |  | 0.801 | 0.701 | 0.649 |

AUC denotes area under receiver operating characteristic curve; EBV denotes Epstein-Barr virus

*Defined as titers ≥ 1:5.
